# Haptoglobin Duplicon, Hemoglobin, and Vitamin C: Analyses in the British Women's Heart and Health Study and Caerphilly Prospective Study

**DOI:** 10.1155/2014/529456

**Published:** 2014-11-30

**Authors:** Philip A. I. Guthrie, Mohammad R. Abdollahi, Tom Gaunt, Debbie A. Lawlor, Yoav Ben-Shlomo, John Gallacher, George Davey Smith, Ian N. M. Day, Santiago Rodriguez

**Affiliations:** ^1^Bristol Genetic Epidemiology Laboratory, School of Social and Community Medicine, University of Bristol, Oakfield House, Oakfield Grove, Bristol BS8 2BN, UK; ^2^MRC Integrative Epidemiology Unit (IEU), University of Bristol, Bristol BS8 2BN, UK; ^3^School of Social and Community Medicine, University of Bristol, Bristol BS8 2PS, UK; ^4^Institute of Primary Care & Public Health, Cardiff University School of Medicine, Cardiff CF14 4XN, UK

## Abstract

*Background*. Haptoglobin acts as an antioxidant by limiting peroxidative tissue damage by free hemoglobin. The haptoglobin gene allele Hp2 comprises a 1.7 kb partial duplication. Relative to allele Hp1, Hp2 carriers form protein multimers, suboptimal for hemoglobin scavenging. *Objective*. To examine the association of haptoglobin genotype with a range of phenotypes, with emphasis on vitamin C and hemoglobin levels. *Methods*. We applied a quantitative PCR assay for the duplication junction to two population cohorts including 2747 British women and 1198 British men. We examined the association of haptoglobin duplicon copy number with hemoglobin and vitamin C and used the copy number to complete a phenome scan. *Results.* Hemoglobin concentrations were greater in those with Hp2,2 genotype, in women only (Hp1,1 13.45 g/dL, Hp1,2 13.49 g/dL, Hp2,2 13.61 g/dL; *P* = 0.002), though statistically there was no evidence of a difference between the sexes (*z* value = 1.2, *P* = 0.24). Haptoglobin genotype was not associated with vitamin C or any other phenotype in either cohort. *Conclusions*. Our results do not support association of haptoglobin genotype with vitamin C or with other phenotypes measured in two population cohorts. The apparent association between haptoglobin genotype and hemoglobin in the women's cohort merits further investigation.

## 1. Introduction

The haptoglobin gene* HP* exists in the human population in two forms: Hp1 and Hp2 (throughout this paper the classic haptoglobin duplicon nomenclature will be used, i.e., Hp1 refers to the allele without the duplication and Hp2 refers to the allele with duplication). Hp1 is the less frequent allele in Europeans. Hp1,1 refers to the homozygote for the Hp1 allele, and Hp2,2 refers to the homozygote for the Hp2 allele. Exons 5 and 6 of Hp2 represent duplication of a 1.7 kb segment containing exons 3 and 4 of Hp1 [1]. The protein product for Hp1,1 forms a dimer, whereas the products of Hp1,2 or Hp2,2 are multimers of varying size and complexity, greater in the Hp2,2 genotype. The principal function of haptoglobin, which is normally present at a greater than 400-fold molar excess compared with free hemoglobin [2], is to scavenge free hemoglobin [3] which has been liberated into the plasma by intravascular haemolysis. The haptoglobin/hemoglobin complex is cleared from the bloodstream by circulating monocytes or in the liver by Kuppfer cells, and the heme iron is recycled. At sites of tissue damage, the complex is cleared by macrophages.

The range of possible forms of haptoglobin, from dimer to large multimer depending on genotype, results in a gene product with a range of properties and potentially complex interactions. For example, although the hemoglobin-binding capacity of Hp2,2 is* lower* than that of Hp1,1 [4], the Hp2,2-hemoglobin complex binds to CD163 (the haptoglobin receptor expressed on macrophage and monocyte cell surfaces) with a 10-fold* higher* affinity than the Hp1,1-hemoglobin complex [5].

The aim of this study was to examine the association of haptoglobin genotype with a wide range of phenotypes. This is justified on the basis of the hypothesised antioxidant properties of haptoglobin and claims for the involvement of antioxidation in a wide range of health related outcomes. However, within this broad phenome scan, we had three specific prior hypotheses.

1. Higher levels of vitamin C would be found in individuals not carrying the Hp2 allele because haptoglobin is involved in redox reaction, which should affect vitamin C levels [6] and, as described above, is intimately involved with the scavenging of circulating hemoglobin.
2. Higher total levels of hemoglobin would be found in individuals carrying the Hp2 allele because of less efficient clearance. This hypothesis was based on the specific high affinity protein interaction of haptoglobin with hemoglobin and reports of haptoglobin genotype association with hemoglobin level in malaria [7].

## 2. Materials and Methods

We developed a novel high-throughput liquid-phase assay for detection of the* Hp* duplicon, based on fluorescence ratiometry between junction and reference polymerase chain reaction (PCR) amplicons using a melt curve analysis [8]. We used this assay to genotype participants in two British cohort studies: the British Women's Heart and Health Study (BWHHS) and the Caerphilly Prospective Study (CaPS) of men. Because of the possibility that copy number variation in* Hp* could have a wide range of effects, relating to its antioxidant properties, we completed a “phenome scan” in these two cohorts, examining a range of blood measures and clinical traits with genotype.

### 2.1. British Women's Heart and Health Study (BWHHS)

BWHHS is a cohort of 4,286 women, who were randomly selected from 23 British towns and who were of the age between 60 and 79 years at enrolment (1999–2001) [9]. At baseline assessment the women completed a questionnaire, a nurse-led health interview, and a physical examination at which fasting blood samples (minimum 8 hours) were taken. Medical records were reviewed for all evidence of past history of cardiovascular disease, type 2 diabetes, and cancer. The women have been followed up with information on new disease events obtained from participant completed questionnaires and a two-year review of their medical records and with mortality data obtained from the UK National Health Service central register.

For further details of BWHHS, including analyte preparation, please see Supplementary Material available online at http://dx.doi.org/10.1155/2014/529456.

### 2.2. Caerphilly Prospective Study (CaPS)

All men from Caerphilly, South Wales, and surrounding villages of age between 45 and 59 years were contacted and invited to take part in a health survey, resulting in 2512 (89% response rate) men participating in the baseline examination that was undertaken between 1979 and 1983 (Phase I). Phase II (1984–1988) consisted of those from Phase I who had been recontacted, together with an additional group who had since moved into the study area (Phase II *n* = 2,398). The examination consisted of a standard medical history, venepuncture for biochemical and hormonal assays, and physical examination including weight, height, blood pressure, and 12-lead ECG. Hemoglobin was measured by a modification of the Dacie and Lewis method using a dedicated hemoglobinometer. Vitamin C was not measured. Subjects were defined as having diabetes (type 2 unless otherwise specified) if they had been previously diagnosed by their general practitioner or if their fasting glucose concentrations were greater than or equal to 7.8 mmoL/L. Other phenome variables were measured as described previously [10]. Of the participants for whom DNA was available, 1198 had complete data on the combination of CHD, type 2 diabetes (based on self-reported diagnosis from questionnaires), and haptoglobin genotype, and so all our analyses were conducted on 1198 men.

#### 2.2.1. Genotyping Assay

The* Hp* junction region and a reference sequence in* TP53* not subject to copy number variation were coamplified by PCR. Reactions were performed using 20 ng genomic DNA as template in 384-well plates. Following 33-cycle PCR optimised to preserve ratio of amplification of test and reference, the ratio of test to reference was determined by fluorescence quantification of Eva Green binding during real time melting of the amplicons in a 384-well LightTyper (Roche Diagnostics); test and reference amplicons were designed to melt at different temperatures [8]. Validations included typing of genotype known DNAs (gift of Dr. Werner Koch (German Heart Centre, Lazarettstrasse 36, D-80636, Munich) and Dr. Joris Delanghe (Department of Laboratory Medicine, University Hospital Ghent, Belgium)), comparison of our ratiometric assay with the assay described by Koch et al. [11], and evaluation of the cluster quality differentiating the clusters of ratios representing 1 or 2 copies of the* HP* duplicon junction. The absence of a target amplicon first derivative fluorescence peak but presence of a reference peak signified zero copies of the duplicon junction.

## 3. Statistics

Linear (for continuous traits) and logistic (for binary traits) regression was used to examine the association of genotype with phenotypes. Where traits were positively skewed (triglycerides, glucose, insulin), we used a natural logarithm transformation in these regression analyses and then back-transformed for table presentation. The *P* value, with Bonferroni correction, for the phenome scan of the BWHHS data was set at (0.05/31 = 0.0016) and of the CaPS data was set at (0.05/24 = 0.0021).

## 4. Results

Table 1 summarises genotype and allele frequency data for the two cohorts.

Genotype and allele frequencies were similar in the two studies.

Table 2 shows the results of tests for association between haptoglobin genotype and vitamin C concentration, hemoglobin level, and CHD, for BWHHS and CaPS (from phase 2). Haptoglobin genotype is associated with hemoglobin level in BWHHS (hemoglobin concentration increasing with number of Hp2 alleles) but not with CHD or vitamin C concentration. Haptoglobin genotype was not associated with coronary disease or hemoglobin concentration in CaPS. Though an association with haemoglobin was observed in BWHHS and not in CaPS, there was no statistical evidence that the association differed between these two studies (*P* value from *z*-test = 0.24).

## 5. Validation Results

Table 3 compares* HP* duplicon genotyping results using the assay of Koch et al. [11] and using our ratiometric assay (amplification ratio control system—ARCS). Genotyping was fully concordant between the two methods apart from Hp1,2 (96% concordance).

The stated* HP* CNV genotypes of the control samples provided by Dr. Werner Koch and Kr. Joris Delanghe were in full agreement with those determined by ARCS (data not shown).

Figure 5 in [8] shows an ARCS plot of reference gene peak height on the *x*-axis against* HP* gene CNV peak height on the *y*-axis, for 85 samples. Hp1,1 Hp1,2, and Hp2,2 are seen to be separated into three distinct clusters radiating from the origin, providing good discrimination among the three genotypes.

Supplementary Data Tables 1 and 2 show the associations of haptoglobin genotype with phenome scan traits in BWHHS and in CaPS, respectively. Haptoglobin genotype was not associated with any phenotypes in either cohort, except for the association with hemoglobin in BWHHS as discussed above.

Supplementary Data Table 3 shows the haptoglobin genotype and allele frequencies by quartile of vitamin C distribution in BWHHS. No associations between genotype/allele frequency and vitamin C distribution were found.

## 6. Discussion

We examined the association between haptoglobin genotype and clinical traits in two British cohorts, with prior hypotheses for association with diabetic CHD, hemoglobin, and vitamin C level. Evidence of association with CHD, diabetic CHD, or vitamin C was lacking. Though haptoglobin genotype did show nominal association with hemoglobin, this was only in BWHHS and was not replicated in CaPS, though statistically the association was consistent between the two studies. Furthermore, despite the hypothesised antioxidant properties of haptoglobin and claims for the involvement of antioxidation in a wide range of health related outcomes, we found no compelling evidence that haptoglobin genotype was associated with a large number of phenotypes examined in our phenome scans (Supplementary Tables 1 and 2).

### 6.1. Haptoglobin Genotype and Vitamin C Status

We did not observe an association of haptoglobin genotype with plasma vitamin C concentrations. A small study of Chinese males (*N* = 50) [12] reported that individuals with Hp2,2-genotype had lower (by 31%) vitamin C concentrations than those with other genotypes (*P* < 0.01) but stated that there was no such association in females (*N* = 60). It has also been reported [13] that overall haptoglobin concentrations are lower in males than females, resulting in lower stability of vitamin C in males. Additionally, Langlois et al. [6] noted that, in participants consuming a vitamin C-poor diet, those with the Hp2,2 genotype were most likely to develop vitamin C deficiency. Similarly, Cahill and El-Sohemy found that Hp2,2 participants had lower vitamin C concentrations when reported dietary vitamin C was low, but not when it met the recommended daily allowance [14]. These reports, together with the observations of Ness et al. [15] who showed a mean plasma vitamin C level in European British females of approximately 1.35 times the level found in males, suggest that our results for the entirely female BWHHS cohort may be a reflection of the vitamin C replete status of a large fraction of the participants [16]. When our data were divided into quartiles by vitamin C level (Supplementary Data Table 3) to test the hypothesis that genotype effect might only be apparent in low vitamin C quartiles, we also found no association.

### 6.2. Haptoglobin Genotype and Hemoglobin Levels

Our data suggest an association of haptoglobin genotype with hemoglobin level in BWHHS but not in CaPS. The difference in the sexes of the two cohorts may partially account for this result, since in several studies, hemoglobin concentration was found to be higher in males compared with females [17–19], but we found no statistical evidence that results did differ between the two cohorts. Alternatively, the lack of association between haptoglobin genotype and hemoglobin concentration in the CaPS cohort may be a type-2 error. Prior biological knowledge of the high affinity specific interaction of haptoglobin with free hemoglobin (its main role) [20], together with a publication showing an association of the severity of anemia with haptoglobin genotype in patients infected with malaria [21] and on reporting an association of free hemoglobin level with haptoglobin genotype [12], led us to hypothesise that* HP* would be associated with hemoglobin in the direction that we observed. Given the complex network of regulation and interactions, direction of effect is difficult to predict. We observe higher hemoglobin in the Hp2,2 group in BWHHS, contrasting with studies in malaria-endemic areas where Hp2,2 (additively) associates with lower hemoglobin levels in children during the malaria season and healthy Hp1,1 subjects display approximately 50% higher free hemoglobin levels in their plasma [12, 21].

Sex differences of iron turnover, as exemplified by protection of* HFE* gene homozygous mutant females from clinical hemochromatosis [22], the higher serum iron concentration found in Hp2,2 men but not Hp2,2 women, and the higher vitamin C concentration found in women [23] may all be important factors influencing effects of haptoglobin genotype.

In summary, our studies found no association between haptoglobin genotype and vitamin C status. An association was found between haptoglobin genotype and hemoglobin concentration in the BWHHS cohort (older women), but not in CaPS (middle-aged men). However, given the large number of associations examined here and some evidence of inconsistency of direction of association of* HP* with hemoglobin in the literature, further replication of this finding is required before we can claim that this association is robust. A wider systems approach will be necessary to simultaneously resolve effects in relation to sex, redox status, free hemoglobin level, total hemoglobin level, and oxygen carriage, with potentially more major effects in the presence of large challenges to homeostasis such as those of malaria, diabetes, or other diseases.

## Supplementary Material

Tables 1 and 2 show association of haptoglobin genotype with phenome traits for the BWHHS and CaPS cohorts, respectively. Table 3 details counts and frequencies for genotypes and alleles in the BWHHS cohort, divided into quartiles by plasma vitamin C distribution. The supplementary material concludes with a summary of how blood samples were collected and analysed for vitamin C and hemoglobin, in the BWHHS cohort.

## Figures and Tables

**Table 1:** Haptoglobin genotype and allele frequency for BWHHS and CaPS cohorts.

Genotype/Allele	Number (%) [expected number]			
	BWHHS		CaPS	
Hp1,1	417 (13.3)	[416.2]	204 (14.4)	[210.9]
Hp1,2	1447 (46.3)	[1448.5]	685 (48.4)	[671.2]
Hp2,2	1261 (40.3)	[1260.2]	527 (37.2)	[533.9]
Hp1	2281 (36.5)		1093 (38.6)	
Hp2	3969 (63.5)		1739 (61.4)	

	BWHHS		CaPS	

HWE chi^2^, 1df (*P*)	0.0 (1.00)		0.6 (0.44)	

Test for difference in allele frequencies between the two cohorts (Chi-square = 3.52, *P* = 0.06) (N.B. totals for those with valid HP CNV genotype data but not necessarily overlapping other phenotypes).

**Table 2:** Association of *HP* CNV genotype with vitamin C and hemoglobin for BWHHS and CaPS cohorts.

		Means (SD) or *N* (%) of traits by genotype			*P* trend
		Hp1,1	Hp1,2	Hp2,2	(1df)
Continuous outcomes: means (SD)					
BWHHS	Vitamin C (*µ*mol/L)	43.53 (27.47)	44.03 (28.75)	42.91 (28.26)	0.51
CaPS	Vitamin C	N/A	N/A	N/A	N/A
BWHHS	Hb (g/dL)	13.45 (1.00)	13.49 (1.14)	13.61 (1.00)	0.002
CaPS	Hb (g/dL)	14.87 (1.35)	14.94 (1.05)	14.91 (1.03)	0.818

Hb: hemoglobin.

**Table 3:** Comparison of *HP* duplicon genotyping results using Koch assay and using Amplification Ratio Control System (ARCS) assay for 64 data points.

	Hp CNV genotype			
	Hp1,1	Hp1,2	Hp2,2	Fail
Koch assay (*n*)	9	25	27	3
ARCS assay (*n*)	9	24	27	4
