# Supplementary material for: Haptoglobin Duplicon, Hemoglobin, and Vitamin C: Analyses in the British Women's Heart and Health Study and Caerphilly Prospective Study
Source: Dis Markers. 2014 Nov 30;2014:529456. doi: 10.1155/2014/529456 (PMC4265517; doi:10.1155/2014/529456)
Supplement: Supplementary file 1 — Tables 1 and 2 show association of haptoglobin genotype with phenome traits for the BWHHS and CaPS cohorts, respectively. Table 3 details counts and frequencies for genotypes and alleles in the BWHHS cohort, divided into quartiles by plasma vitamin C distribution. The supplementary material concludes with a summary of how blood samples were collected and analysed for vitamin C and hemoglobin, in the BWHHS cohort. [file 529456.f1.docx]

Supplementary Data Table 1.

# Association of haptoglobin genotype with phenome scan traits, in BWHHS cohort.

_____________________________________________________________________

**Means (SD) or N (%) of traits by P trend**

**genotype (1df)**

**Hp1,1 Hp1,2 Hp2,2**

**_____________________________________________________________________**

**Continuous outcomes means (SD)**

Vitamin C (μmol/l) 43.53 (27.47) 44.03 (28.75) 42.91 (28.26) 0.51

Vitamin C* 33.78 34.47 32.79 0.39

Age (years) 69.03 (5.34) 68.89 (5.59) 68.60 (5.43) 0.13

BMI (kg/m^2^) 27.68 (4.79) 27.52 (4.94) 27.67 (5.09) 0.81

WHR*100 81.84 (6.38) 81.88 (6.80) 82.12 (7.03) 0.39

SBP (mmHg) 147.51 (25.16) 147.08 (25.70) 146.88 (24.95) 0.69

DBP (mmHg) 79.32(11.61) 79.45 (11.65) 79.64 (11.74) 0.60

Pulse rate (per minute) 71.87(13.31) 71.09(12.57) 71.38 (12.69) 0.77

FEV_1_ (L) 2.01 (0.50) 1.97 (0.54) 1.99 (0.53) 0.94

Total Hemoglobin 13.45 (1.00) 13.49 (1.13) 13.61 (1.00) 0.002

Mean Cell Hemoglobin 29.53 (1.68) 29.53 (1.94) 29.59 (1.73) 0.49

Mean Cell Volume 91.27 (4.77) 91.27 (5.53) 91.41 (4.88) 0.59

Red Cell Count (x10^6^/ml) 4.56 (0.36) 4.57 (0.39) 4.61 (0.36) 0.01

Monocyte Count (x10^3^/ml) 0.33 (0.13) 0.33 (0.15) 0.34 (0.14) 0.07

Total Chol (mmol/l) 6.60 (1.28) 6.62 (1.20) 6.66 (1.22) 0.32

LDLc (mmol/l) 4.07 (1.03) 4.16 (1.08) 4.17 (1.13) 0.17

HDLc (mmol/l) 1.66 (0.48) 1.62 (0.44) 1.64 (0.46) 0.91

Triglycerides (mmol/l)* 1.68 1.65 1.70 ( 0.52

Insulin (pmol/l)* 7.03 6.96 7.03 0.851

Glucose (mmol/l)* 6.05 5.93 5.99 0.66

D-dimer (µg/l) N/A N/A N/A N/A

Northing for area of 398.2 405.8 404.5 0.69

birth (Km North of the (179.4) (179.2) (175.7)

National grid origin^†^)

Easting for area of birth 404.0 399.3 401.6 0.92

(Km East of the (103.1) (97.6) (99.3)

National grid origin^†^)

**Binary outcomes n (%)**

Adult manual social 207 (54.6) 663 (52.54) 567 (51.27) 0.52

class n (%)

Child manual social 312 (82.3) 1012 (80.19) 879 (79.5) 0.49

class n (%)

Ever smoked n (%) 167 (44.1) 562 (44.6) 487 (44.1) 0.97

Obese n (%) 101 (27.0) 323 (25.9) 284 (25.8) 0.90

Diabetes n (%) 48 (12.7) 119 (9.4) 118 (10.7) 0.18

Gall bladder disease n (%) 32 (9.3) 95 (8.4) 93 (9.4) 0.66

Self-report DVT 20 (5.3) 79 (6.3) 54 (4.9) 0.34

Self-report PE 3 (0.8) 28 (2.2) 16 (1.4) 0.12

Self-report DVT or PE 21 (5.6) 94 (7.5) 60 (5.4) 0.10

______________________________________________________________________

BMI: body mass index; WHR: waist/hip ratio; SBP: systolic blood pressure; DBP: diastolic blood pressure; FEV_1_: forced expiratory volume in 1 second; LDLc: low density lipoprotein cholesterol; HDLc: high density lipoprotein cholesterol; DVT: deep vein thrombosis; PE: pulmonary embolus; NB the categories of DVT or PE do not have totals that equal DVT plus PE since several individuals had both and only count once (one person) in the combined category.

* Mean of logged variable.

^†^ National grid origin is near to Scilly Isles, which is one of the furthest South Westerly points of Britain. Higher values for Northing represent more Northerly areas and higher values for Easting more Easterly areas.

Supplementary Data Table 2.

Association of haptoglobin genotype with phenome scan traits, in CaPS cohort.

_____________________________________________________________________

**Hp1,1 Hp1,2 Hp2,2 P trend**

**(1df)**

**_____________________________________________________________________**

**Continuous outcomes means (SD)**

Vitamin C N/A N/A N/A N/A

Age (years) 56.62(4.50) 56.73(4.49 56.82(4.40) 0.59

BMI (kg/m^2^) 26.84(3.58) 26.55(3.79 26.41(3.46) 0.20

WHR*100 93.09(5.21) 93.04(7.15) 93.03(7.67) 0.93

SBP (mmHg) 143.45(21.55) 145.54(23.55) 146.72(22.23) 0.11

DBP (mmHg) 83.67(11.27) 83.94(12.19) 85.53(11.31) 0.03

Pulse rate (per minute) 65.21(11.42) 65.22(10.96) 65.35(11.61) 0.86

FEV_1_(L) 2.78(0.65) 2.75(0.70) 2.71(0.68) 0.21

Total hemoglobin 14.87(1.35) 14.94(1.05) 14.91(1.03) 0.82

Mean RBC Hb 30.60(2.06) 30.94(1.80) 30.86(1.85) 0.30

Mean RBC volume 91.78(5.19) 92.34(4.78) 92.08(4.82) 0.78

RBC count (x10^3^/ml) 4.87(0.39) 4.84(0.37) 4.84(0.36) 0.61

Monocyte count (x10^3^/ml) 0.59(0.16) 0.58(0.16) 0.61(0.17) 0.15

Total chol (mmol/l) 5.64(1.02) 5.55(1.00) 5.70(0.94) 0.23

LDLc (mmol/l) 3.80(1.16) 3.82(0.99) 3.82(0.96) 0.85

HDLc (mmol/l) 1.01(0.25) 1.03(0.26) 1.03(0.25) 0.55

Triglycerides (mmol/l)* 1.68 1.67 1.67 0.78

Insulin (pmol/l)* 6.05 5.64 5.70 0.33

Glucose (mg/dl)* 52.98 52.46 52.98 0.89

D-dimer (µg/l) 81.01(61.09) 88.65(85.53) 77.25(51.11) 0.20

Northing/Easting N/A N/A N/A N/A

**Binary outcomes n(%)**

Father’s manual soc class 119(90.15) 387(87.16) 286(88.00) 0.94

Manual social class 117(65.73) 396(66.22) 280(65.12) 0.88

Ever smoked 147(82.58) 481(80.43) 348(80.93) 0.69

Obese 34(19.43) 89(15.11) 48(11.27) 0.03

Diabetes 14(7.91) 43(7.24) 34(7.98) 0.95

Gall bladder disease N/A N/A N/A N/A

Self-report DVT N/A N/A N/A N/A

Self-report PE N/A N/A N/A N/A

Self-report DVT or PE N/A N/A N/A N/A

_____________________________________________________________________

BMI: body mass index; WHR: waist: hip ratio; SBP: systolic blood pressure; DBP: diastolic blood pressure; FEV_1_: forced expiratory volume in 1 second; LDLc: low density lipoprotein cholesterol; HDLc: high density lipoprotein cholesterol; DVT: deep vein thrombosis; PE: pulmonary embolus; NB the categories of DVT or PE do not have totals that equal DVT plus PE since several individuals had both and only count once (one person) in the combined category.

^*^Mean of logged variable.

# Supplementary Data Table 3.

# Hp genotype and allele counts and frequencies (in brackets) by quarters of plasma vitamin C distribution in BWHHS cohort

_____________________________________________________________________

**Genotype / Quarter of vitamin C distribution (range, μmol/l), number**

**Allele**

**Lowest** **2^nd^** **3^rd^ Highest**

**(0.0-21.42) (21.42-39.79) (39.87-60.78) (60.82-190.47)**

**N = 686 N = 687 N = 687 N = 687**

**_____________________________________________________________________**

Hp1,1 89 (12.97) 99 (14.41) 94 (13.68) 97 (14.12)

Hp1,2 312 (45.48) 308 (44.83) 327 (47.60) 315 (45.85)

Hp2,2 285 (41.54) 280 (40.76) 266 (38.72) 275 (40.03)

Hp1 490 (35.71) 506 (36.83) 515 (37.48) 509 (37.04)

Hp2 882 (64.29) 868 (63.17) 859 (62.52) 865 (62.95)

HWE χ^2^ = 0.06 χ ^2^ = 0.91 χ ^2^ = 0.17 χ ^2^ = 0.2

p = 0.81 p = 0.34 p = 0.68 p = 0.65

_____________________________________________________________________

Supplementary Information

*British Women’s Heart and Health Study (BWHHS).*

The women were defined as having type 2 diabetes at baseline on the basis of reporting this in the questionnaire or identification in medical record reviews or if they had a fasting blood glucose equal to or greater than 7mmol/l. If a participant indicated they had diabetes in the questionnaire only and it was unclear which type, they were defined as having type 2 diabetes if they were older than 30 years at diagnosis (or with an unknown age at diagnosis) [1]. Baseline type 2 diabetes was used to stratify women in the analyses used to compare gene-CHD associations in those with and without type 2 diabetes.

Venous blood samples were taken after a minimum 8-hour fast onto EDTA. The sample was centrifuged within 2h of being drawn from the participant and 0.5ml of plasma was removed in a Sarstedt 2ml glass tube and mixed with 0.5ml 100g metaphosphoric acid/L; assays were then snap frozen on dry ice and on return to the laboratory frozen at -80° C before analysis.

For measurement of plasma vitamin C concentration, samples were vortexed after thawing and spun in a microcentrifuge for 30min at 4° C at 5000*g*. The centrifugation was repeated and the supernatant passed through a 40,000 molecular filter at 2000*g* to remove traces of protein. Standards of pure ascorbate were treated in a similar manner. All samples and standards were used for analysis of ascorbate by high performance liquid chromatography[2;3] This was undertaken using a Jasco UK (Great Dunmow, Essex) liquid chromatograph fitted with a reverse phase column. The mobile phase consisted of 839ml acetonitrile/ 16ml15mM KH_2_PO_4_ buffer/ 0.1 glacial acetic acid and the peaks were detected using uv absorption at 254m. The amount of ascorbate per sample was calculated against the diluted solutions of stock ascorbate and an internal standard of uric acid.

Hemoglobin was assayed in local haematology departments in each of the towns within 24 hours of the blood being taken, using the standard Coulter counter method on EDTA blood. Details of the assessment of all other phenotypes used in the phenome scan have been previously reported [4].

Of the women originally enrolled in the study, 3125 (72.9%) had valid *HP* CNV genotype data. Of these 3125*,* 2747 had complete data on the combination of CHD, type 2 diabetes, vitamin C, Hb and Hp genotype, and were of European ancestry. Thus, all our analyses are conducted on these 2747 women with complete data up to September 2007.

Reference List

1. Andersen AF, Carson C, Watt HC, Lawlor DA, Avlund K, Ebrahim S. (2008) Life-course socio-economic position, area deprivation and Type 2 diabetes: findings from the British Women's Heart and Health Study. Diabet Med 25:1462-8

2. (1984) Caerphilly and Speedwell collaborative heart disease studies. The Caerphilly and Speedwell Collaborative Group. J Epidemiol Community Health 38:259-62

3. Rice-Evans, Diplock CA, Symons MCR. Laboratory techniques in biochemistry and molecular biology. Elsevier, 1991:185-206pp.

4. Lawlor DA, Bedford C, Taylor M, Ebrahim S. (2003) Geographical variation in cardiovascular disease, risk factors, and their control in older women: British Women's Heart and Health Study. J Epidemiol Community Health 57:134-40
